# Functional Analysis of Mouse G6pc1 Mutations Using a Novel *In Situ* Assay for Glucose-6-Phosphatase Activity and the Effect of Mutations in Conserved Human G6PC1/G6PC2 Amino Acids on G6PC2 Protein Expression

**DOI:** 10.1371/journal.pone.0162439

**Published:** 2016-09-09

**Authors:** Kayla A. Boortz, Kristen E. Syring, Lynley D. Pound, Yingda Wang, James K. Oeser, Richard M. O’Brien

**Affiliations:** Department of Molecular Physiology and Biophysics, Vanderbilt University School of Medicine, Nashville, Tennesse, 37232, United States of America; Medical Clinic, University Hospital Tuebingen, GERMANY

## Abstract

Elevated fasting blood glucose (FBG) has been associated with increased risk for development of type 2 diabetes. Single nucleotide polymorphisms (SNPs) in *G6PC2* are the most important common determinants of variations in FBG in humans. Studies using *G6pc2* knockout mice suggest that G6pc2 regulates the glucose sensitivity of insulin secretion. *G6PC2* and the related *G6PC1* and *G6PC3* genes encode glucose-6-phosphatase catalytic subunits. This study describes a functional analysis of 22 non-synonymous *G6PC2* SNPs, that alter amino acids that are conserved in human G6PC1, mouse G6pc1 and mouse G6pc2, with the goal of identifying variants that potentially affect G6PC2 activity/expression. Published data suggest strong conservation of catalytically important amino acids between all four proteins and the related G6PC3 isoform. Because human G6PC2 has very low glucose-6-phosphatase activity we used an indirect approach, examining the effect of these SNPs on mouse G6pc1 activity. Using a novel *in situ* functional assay for glucose-6-phosphatase activity we demonstrate that the amino acid changes associated with the human *G6PC2* rs144254880 (Arg79Gln), rs149663725 (Gly114Arg) and rs2232326 (Ser324Pro) SNPs reduce mouse G6pc1 enzyme activity without affecting protein expression. The Arg79Gln variant alters an amino acid mutation of which, in G6PC1, has previously been shown to cause glycogen storage disease type 1a. We also demonstrate that the rs368382511 (Gly8Glu), rs138726309 (His177Tyr), rs2232323 (Tyr207Ser) rs374055555 (Arg293Trp), rs2232326 (Ser324Pro), rs137857125 (Pro313Leu) and rs2232327 (Pro340Leu) SNPs confer decreased G6PC2 protein expression. In summary, these studies identify multiple *G6PC2* variants that have the potential to be associated with altered FBG in humans.

## Introduction

Elevated fasting blood glucose (FBG) has been associated with increased risk for the development of type 2 diabetes (T2D) and cardiovascular associated mortality (CAM) [1–3]. Previous studies have shown that an increase in FBG of 9–18 mg/dl is associated with a ~30% increased risk of CAM [2]. Conversely, a reduction in FBG of ~9 mg/dl is associated with a 25% reduction in risk of CAM [3]. Multiple groups have performed genome wide association studies (GWAS) in an effort to identify genes associated with variations in FBG. These studies have identified SNPs that effect glucose homeostasis in over 50 loci, many of which are also associated with T2D [4–7]. Notably, the rs560887 single nucleotide polymorphism (SNP) located in the third intron of the *G6PC2* locus has been identified as the strongest common genetic determinant of FBG levels in terms of significance and effect size, accounting for ~1% of total variance in FBG [4,6–11]. Molecular studies have shown that this intronic SNP affects *G6PC2* RNA splicing [12]. Further genetic and molecular analyses of this locus have shown that two common promoter SNPs, rs13431652 and rs2232316, in addition to the intronic rs560887 SNP, are potentially causative and may contribute to the association between *G6PC2* and FBG [12,13].

*G6PC2*, formerly known as *IGRP*, encodes an islet-specific glucose-6-phosphatase catalytic subunit [14–16] that catalyzes the hydrolysis of glucose-6-phosphate (G6P) to glucose and inorganic phosphate (Pi) [11,17]. Studies on *G6pc2* knockout (KO) mice show that G6pc2 is a negative regulator of glucose-stimulated insulin secretion (GSIS) [11,16]. G6pc2 acts by hydrolyzing G6P, thereby increasing glucose cycling and presumably decreasing glycolytic flux [16,18]. As such, G6pc2 opposes the action of the glucose sensor glucokinase [19,20]. Both male and female *G6pc2* KO mice have significantly decreased FBG levels [15,16,21], consistent with GWAS data and molecular studies.

Common variants associated with variations in FBG were thought to account for a low percentage (~10%; Ref. [22]) of total heritable variation, leading to speculation that rare (minor allele frequency <5%), high impact variants undetected by GWAS might account for the remaining 90% of heritability [23]. However, more recent studies have suggested that the combined effects of multiple common variants have the potential to largely account for missing heritability [24–26]. Nevertheless, the identification of high impact rare variants has provided tremendous insight into beta cell biology [27,28]. For example, while common SNPs in the glucokinase (*GCK*) *GCK* locus have modest effects on FBG [8,9], rare inactivating variants in the *GCK* locus have been shown to cause neonatal diabetes mellitus or maturity-onset diabetes in youth while rare activating variants cause hyperinsulinemia [27,28]. Evolutionarily this is logical, as rare variants with significant detrimental effects on health would be selected against and therefore not be propagated in the human population. These data also highlight an important caveat in the interpretation of GWAS data: the effect size of common genetic variants does not necessarily reflect the importance of the gene in relation to the disease or phenotype being studied. As observed with *GCK* and *G6PC2*, despite the greater effect size of common *G6PC2* variants on FBG, deletion of the *Gck* gene in mice is lethal [29] whereas deletion of *G6pc2* results in a mild reduction in FBG [15,16].

Because the identification of high impact rare variants in *GCK* has provided tremendous insight into beta cell biology [27,28], we were interested in identifying variants in *G6PC2* that affect G6PC2 protein expression. We were also interested in identifying variants that potentially have a significant effect on G6PC2 enzyme activity. However, because G6PC2 has much lower enzyme activity than G6PC1, we addressed this second question indirectly by analyzing the effect of 22 non-synonymous *G6PC2* SNPs, that change amino acids that are conserved in human G6PC1, mouse G6pc1 and mouse G6pc2, on G6pc1 enzyme activity using a novel *in situ* functional assay for glucose-6-phosphatase activity.

## Materials and Methods

### Cell Culture

Rat islet-derived 832/13 cells and monkey kidney-derived COS 7 cells were passaged as sub-confluent cultures in RPMI medium supplemented with 10% (vol/vol) fetal bovine serum, 0.05 mM **β**mercaptoethanol, 100 U/ml penicillin and 100 μg/ml streptomycin.

### SNP Databases

Human *G6PC2* SNPs were identified using the UCSC Genome Browser (https://genome.ucsc.edu/), HumSAVR (http://omictools.com/humsavar-tool) or dbSNP (http://www.ncbi.nlm.nih.gov/SNP/) databases.

### G6PC Expression Vector Construction

The construction of plasmids encoding human G6PC2 (accession number NM_021176), mouse G6pc2 (accession number NM_021331), human G6PC1 (accession number NM_000151) and mouse G6pc1 (accession number NM_008061) in the pcDNA3.1D v5-His-TOPO vector with a C-terminal V5-His Tag has been previously described [30,31]. A common SNP, rs492594, switches a valine to a leucine at amino acid (AA) 219 in human G6PC2 [14]. The human G6PC2 sequence designated as wild type (WT) in this study contained a leucine at AA 219.

Human G6PC2:mouse G6pc2 and mouse G6pc2:human G6PC2 chimeras in the pcDNA3.1D v5-His-TOPO vector were constructed by ligating fragments of the respective open reading frames. This was achieved by sub-cloning using a combination of restriction enzyme sites in the pcDNA3.1D v5-His-TOPO vector and the internal Dra I, Stu I and BamH I restriction enzyme sites, which are common to both human G6PC2 and mouse G6pc2. These three sites truncate G6PC2/G6pc2 at AAs 72, 192 and 249, respectively, from the N terminus.

Site-directed mutagenesis was used to change specific codons in human *G6PC2* and mouse *G6pc1*. This was achieved either by using the Quikchange II kit (Agilent Technologies, Santa Clara, CA) or a three-step PCR protocol [32]. DNA sequencing was used to verify all codon changes and the absence of secondary mutations. Two to three independent plasmid preparations were analyzed for each SNP variant described. Plasmids were purified by centrifugation through cesium chloride gradients [32].

### *G6pc1* and *Pklr* Fusion Gene Construction

A bacterial artificial chromosome (BAC) clone (CH230-220J5) containing the entire rat *G6pc1* gene (Accession number AC123346) was purchased from BACPAC Resources, Children's Hospital Oakland Research Institute, Oakland, CA. This clone was digested with Kpn I to isolate a 7319 bp fragment, representing the rat *G6pc1* promoter region between -7253 and +66 [33], that was then ligated into the *Kpn* I digested pGEM7 vector (Promega, Madison, WI). This fragment was then re-isolated, blunted ended using Klenow and ligated into the Xba I—Bgl II, digested and Klenow treated pCAT(An) vector, a gift from Dr. Howard Towle [34]. Fragments of the rat *G6pc1* promoter, representing promoter sequence between -7248 and +62 and -1640 and +62, were then re-isolated from the pCAT(An) plasmid by digestion with Hind III and Xho I and ligated into the Hind III and Xho I digested pGL3 MOD luciferase vector [30].

Two fragments of the rat liver pyruvate kinase (*Pklr*) promoter representing sequences from -206 to +1 and -100 to +1 [35], were generated by PCR reaction using rat genomic DNA as the template in conjunction with the following primers:

(5'-CCCAAGCT(-206)TCTGCAGACAGGCCAAAGGGGATCC-3'),

(5'-CCCAAGCT(-100)TGCTAGCTGGTTATACTTTAAC-3'), and

(5'-CCGCTCGAGA(+1)CCTGCTGTGTCTGTGGGTCTGCT-3'); Hind III and Xho I cloning sites underlined. The PCR fragments generated were digested with Hind III and Xho I, ligated into the Hind III and Xho I digested pGEM7 vector and then sequenced to ensure the absence of polymerase errors. The fragments were then re-isolated from the pGEM7 plasmid and ligated into the Hind III and Xho I digested pGL3 MOD luciferase vector [30].

### RNA Isolation and Quantification

To compare wild type human *G6PC2* and mouse *G6pc2* RNA expression, plasmids encoding human G6PC2 and mouse G6pc2 (3 μg) were transiently transfected in semi-confluent COS 7 cells in 10 cm diameter dishes using the lipofectamine reagent (InVitrogen, Waltham, MA) as previously described [36]. Following transfection, cells were incubated for 18–20 hours in serum-containing medium. RNA was then harvested and purified using the RNAqueous® kit (Ambion, Carlsbad, CA). Gene expression was then quantitated by using the Turbo DNA-free DNAse Treatment Kit (Ambion, Carlsbad, CA) to remove trace genomic DNA followed by cDNA generation using the iScript DNA Synthesis Kit (Bio-Rad, Hercules, CA) and then PCR using the dUTP-containing FastStart SYBR Green Master Mix in conjunction with Uracil-Glycosylase (Roche, Nutley, NJ). PCR products were analyzed by electrophoresis on 1% agarose gels.

The following primer pairs, that recognize non-coding sequences in the pcDNA3.1D v5-His-TOPO vector, were used for the analysis of both human *G6PC2* and mouse *G6pc2* RNA expression:

pcDNA Forward 5’ CCCAAGCTTGGTACCGAGCTCGGATCCAGT

pcDNA Reverse 3’ CCCGTTTAAACTCAATGGTGATGGTGATGATGACCGGTA

The pcDNA forward primer recognizes 5’ untranslated leader sequence in the mRNA. This sequence represents part of the polylinker 3’ of the CMV transcription start site. The pcDNA reverse primer recognizes 3’ untranslated sequence in the mRNA. This sequence represents the region immediately 3’ of the V5 His tag.

Monkey cyclophilin A (*PPIA*) expression was quantitated as an internal control using the following primers:

Monkey *PPIA* Forward 5’-AATGGCACTGGTGGCAAGTC -3’

Monkey *PPIA* Reverse 5’- GCTCCATGGCCTCCACAATA -3’

### Protein Expression, Western blotting and Luciferase Assays

To determine whether wild type and variant proteins were expressed at similar levels, plasmids encoding human G6PC2 and mouse G6pc1 variants (2 μg) were transiently transfected in semi-confluent 832/13 or COS 7 cells in 3.5 cm diameter dishes using the lipofectamine reagent (InVitrogen, Waltham, MA) as previously described [36]. Following transfection, cells were incubated for 18–20 hours in serum-containing medium. Cells were then harvested using 50 mM Tris, pH 8.0, 150 mM NaCl, 5.8 mM PMSF, and 1% NP-40. Protein samples were quantified using the Pierce™ BCA Protein Assay kit (Thermo Fisher Scientific, Waltham, MA). 20 μg of cell extract was electrophoresed on 10% SDS-polyacrylamide gels and the proteins transferred to PVDF membrane (Perkin Elmer, Waltham, MA). Protein expression was determined by immunoblotting with a conjugated mouse monoclonal Anti-V5-horseradish peroxidase (HRP) antibody (1:100–1:5000, InVitrogen, Waltham, MA). A primary anti-beta actin monoclonal antibody (1:10,000, Sigma, St. Louis, MO) with an Anti-Mouse HRP secondary antibody (1:10,000, Promega, Madison, WI) was used to determine beta actin expression as a loading control. HRP activity was assayed using the Pierce™ ECL reagent (Thermo Fisher Scientific, Waltham, MA). Protein expression data were normalized by scanning both V5 and actin signals on Western blots. The ratio of V5 to actin expression obtained with the variants shown was expressed as a percentage relative to the ratio obtained with WT human G6PC2 or mouse G6pc1.

**Fig 1:**
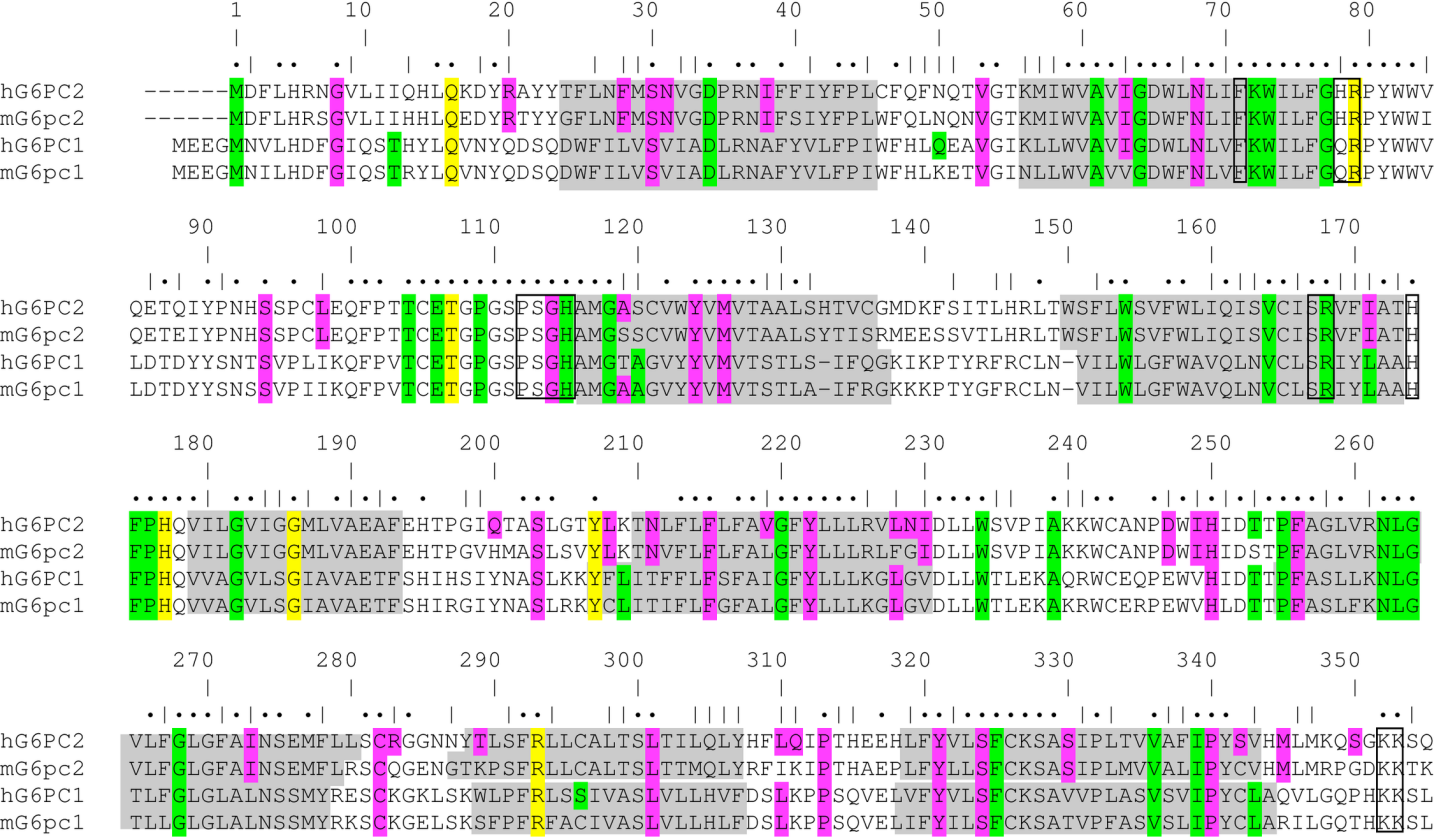
Conservation of Amino Acids Between Human 12, Mouse G6pc2, Human G6PC1 and Mouse G6pc1. Sequence alignment showing the conservation of AAs between human (h) G6PC2, mouse (m) G6pc2, human G6PC1 and mouse G6pc1. Residues highlighted in green represent AAs mutation of which in G6PC1 causes GSD type 1a [40]. Residues highlighted in pink represent AAs that are changed by human *G6PC2* SNPs that were identified using the UCSC Genome Browser (https://genome.ucsc.edu/) and HumSAVR (http://omictools.com/humsavar-tool) databases. Residues highlighted in yellow represent conserved AAs in human G6PC2, mouse G6pc2, human G6PC1 and mouse G6pc1 that are changed by a human *G6PC2* SNP and where mutation in G6PC1 can cause GSD type 1a. Identities are indicated by filled circles and similarities by vertical bars.

The expected sizes of human G6PC2, mouse G6pc2, human G6PC1 and mouse G6pc1 with V5 His tags are 45.60, 45.71, 45.54 and 45.51 kDa. As previously observed, both the human G6PC1 [30] and mouse G6pc1 [31] expression plasmids generate doublets, possibly though the use of alternate methionine start codons (Fig 1).

For fusion gene analyses, semi-confluent 832/13 cells in 3.5 cm diameter dishes were co-transfected with 2 μg of a *G6pc1*- or *Pklr* firefly luciferase fusion gene construct, 0.5 μg of SV40-*Renilla* luciferase (Promega, Madison, WI) and the indicated amount of a wild type or variant G6pc1 expression vector, using the lipofectamine reagent (InVitrogen, Waltham, MA) as previously described [36]. Following transfection, cells were incubated for 18–20 hours in serum-free medium supplemented with 2 or 30 mM glucose. Cells were then harvested using passive lysis buffer (Promega, Madison, WI). Firefly and *Renilla* luciferase activity were assayed using the Dual Luciferase Assay kit (Promega, Madison, WI). To correct for variations in transfection efficiency, the results were calculated as a ratio of firefly to *Renilla* luciferase activity. As indicated in the Table and Figure Legends, results were presented either as this ratio or relative to the ratio obtained with 30 mM glucose or relative to the ratio obtained at 30 mM glucose in the presence of either catalytically dead G6pc1 or WT G6pc1. Fusion gene expression was assessed in multiple transfections using at least two independent preparations of each plasmid, as indicated in the Table and Figure Legends.

### Statistical Analysis

All data were analyzed using the Student’s t-test: two sample assuming equal variance. The level of significance was as indicated (two-sided test).

## Results

### Analysis of Glucose-6-Phosphatase Activity

We have previously shown that glucose-6-phosphate activity is abolished in *G6pc2* KO mouse islets strongly suggesting that G6pc2 has phosphohydrolase activity [16]. However, while several groups have attempted to detect G6P hydrolysis following overexpression of human G6PC2 or mouse G6pc2 [14,37,38], only one group has been successful [39]. Petrolonis et al. demonstrated that the rate of G6P hydrolysis by G6PC2 overexpressed in COS7 cells was 20–40 fold lower than that of G6PC1 [39]. This suggests that there are inherent technical difficulties in demonstrating G6P hydrolysis following overexpression of G6PC2.

Because of the low enzyme activity of G6PC2 in this study we decided to focus on non-synonymous human *G6PC2* SNPs that alter AAs that are conserved in the highly related [14] and much more enzymatically active human G6PC1 and mouse G6pc1 isoforms of the glucose-6-phosphatase catalytic subunit (Fig 1; Table 1). Specifically, we decided to analyze the effect of these *G6PC2* SNPs indirectly by examining their effect on mouse G6pc1 enzyme activity. We hypothesize that *G6PC2* SNPs that affect the function of mouse G6pc2 are highly likely to affect the function of human G6PC2 because of the strong conservation of catalytically important amino acids between these proteins [14]. Supporting this hypothesis is the observation that of the 56 AAs in human G6PC1 mutation of which gives rise to glycogen storage disease (GSD) type 1a [40], 51 are conserved or represent conserved changes in human G6PC2 (S1 Table). Based on this logic, we searched available databases for non-synonymous human *G6PC2* SNPs that alter AAs that are conserved in mouse G6pc2 and the highly related and much more enzymatically active human G6PC1 and mouse G6pc1 isoforms of the glucose-6-phosphatase catalytic subunit. We identified 22 such non-synonymous human *G6PC2* SNPs (Fig 1; Table 1). These SNPs change AAs in a number of different regions of G6PC2 (Table 1), based on the predicted membrane topology of human G6PC1 [41]. We analyzed the effect of these *G6PC2* SNPs indirectly by examining their effect on mouse G6pc1 enzyme activity using a novel *in situ* assay.

**Table 1:** Analysis of the Effect of Amino Acids Changed by Human *G6PC2* SNPs on Human G6PC2 and Mouse G6pc1 Protein Expression and Activity. Amino acids (AAs) changed by human *G6PC2* SNPs that are conserved in human G6PC2, mouse G6pc2, human G6PC1 and mouse G6pc1 were identified using the UCSC Genome Browser (https://genome.ucsc.edu/) and HumSAVR (http://omictools.com/humsavar-tool) databases. The G6PC2 domain affected by each AA change was predicted by comparison with the proposed structure of G6PC1 [41]. The Table shows the effect of these SNPs on G6pc1 enzyme activity based on comparison with wild type (WT) G6pc1 as assessed using a novel *in situ* enzyme assay (Figs 2 & 3). This assay measures the ability of G6pc1 to suppress glucose-stimulated fusion gene expression (Figs 2 & 3). Results for each variant represent the mean ± S.E.M. of 3 experiments using two independent preparations of each expression vector construct in which each experimental condition was assayed in triplicate. While all of these *G6PC2* SNPs change AAs that are conserved in G6PC1, mutation of some of these AAs in G6PC1 causes GSD type 1a [40]. In each case the AA associated with GSD type 1a is shown in parentheses. In each case the *G6PC2* SNP changes the AA to one distinct from that associated with GSD type 1a. For simplicity and comparisons between human G6PC2 and mouse G6pc1 the AAs in mouse G6pc1 are numbered based on the position of the equivalent conserved AA in human G6PC2 (Fig 1). N.D., not determined; N.C., no change. **, these residues have been associated with variations in FBG in healthy individuals who do not have diabetes [50,52,53].

*hG6PC2* SNP	Base #	AA#	GSD Type1a Mutation	Domain Location	hG6PC2 Expression	mG6pc1 Expression	% WT mG6pc1 Activity	P Value
rs368382511	GGA23GAA	Gly8Glu	No	N terminus	Decreased	Decreased	90.13 ± 2.97	0.03
rs372008743	CAG48CAT	Gln16His	Yes (Arg)	N terminus	N.D.	N.C.	110.04 ± 1.71	0.001
rs142189264	TCC89TTC	Ser30Phe	No	In membrane 1	N.D.	N.C.	67.22 ± 1.39	0.00002
rs375874967	GTT157ATT	Val53Ile	No	In loop	N.D.	N.C.	109.98 ± 2.07	0.01
rs199682245	AAT203ATT	Asn68Ile	No	In membrane 2	N.D.	N.C.	79.45 ± 4.46	0.01
rs144254880	CGA236CAA	Arg79Gln	Yes (His or Cys)	In membrane 2	N.D.	N.C.	52 ± 1.07	0.000001
rs371234742	ACA320AGA	Thr107Arg	Yes (Ile)	In loop	N.D.	N.C.	84.25 ± 4.99	0.03
rs149663725	GGC340CGC	Gly114Arg	No	In loop	N.D.	N.C.	53.32 ± 0.88	0.000001
rs187707963	TAT371TGT	Tyr124Cys	No	In membrane 3	N.D.	N.C.	104 ± 0.92	0.01
rs367930047	ATG376GTG	Met126Val	No	In membrane 3	N.D.	N.C.	103.96 ± 5.75	0.53
rs138726309	CAT529TAT	His177Tyr**	Yes (Pro)	In membrane 5	Decreased	N.C.	92 ± 2.69	0.03
rs201094274	AGT609AGA	Ser203Arg	No	In loop	N.D.	N.C.	115.33 ± 3.85	0.003
rs2232323	TAC620TCC	Tyr207Ser**	Yes (Cys)	In loop	Decreased	N.C.	88.19 ± 6.53	0.02
rs139587795	TTC644TCC	Phe215Ser	No	In membrane 6	N.D.	N.C.	105 ± 5.09	0.42
rs375527806	TAC664CAC	Tyr222His	No	In membrane 6	N.D.	N.C.	94.59 ± 2.37	0.08
rs147360987	CAC748TAC	His250Tyr	No	In loop	N.D.	N.C.	94.04 ± 5.08	0.31
rs150538801	TTT766CTT	Phe256Leu	No	In loop	N.D.	N.C.	78.96 ± 1.95	0.0004
rs374055555	CGG877TGG	Arg293Trp	Yes (Cys)	In membrane 8	Decreased	Decreased	83.55 ± 2.56	0.003
rs141041285	TTG902TCG	Leu301Ser	No	In membrane 8	N.D.	N.C.	86.94 ± 5.85	0.02
rs137857125	CCG938CTG	Pro313Leu	No	In loop	Decreased	N.C.	83 ± 5.48	0.04
rs2232326	TCT970CCT	Ser324Pro**	No	In membrane 9	Decreased	N.C.	58.72 ± 6.27	0.03
rs2232327	CCC1019CTC	Pro340Leu	No	In membrane 9	Decreased	Decreased	55.31 ± 4.93	0.001

**, indicates SNPs that have been association with variations in fasting plasma glucose in non-diabetic individuals.

### Characterization of a Novel Assay for the Measurement of Glucose-6-Phosphatase Activity *In Situ*

Because the activity of G6pc1 appears to be regulated by unknown factors [42], it is unclear whether glucose-6-phosphatase activity assayed *in vitro* truly reflects activity in intact cells. Therefore, before beginning the functional analysis of non-synonymous human *G6PC2* SNPs on mouse G6pc1 activity, we first developed a novel assay for the measurement of glucose-6-phosphatase activity *in situ*.

**Fig 2:**
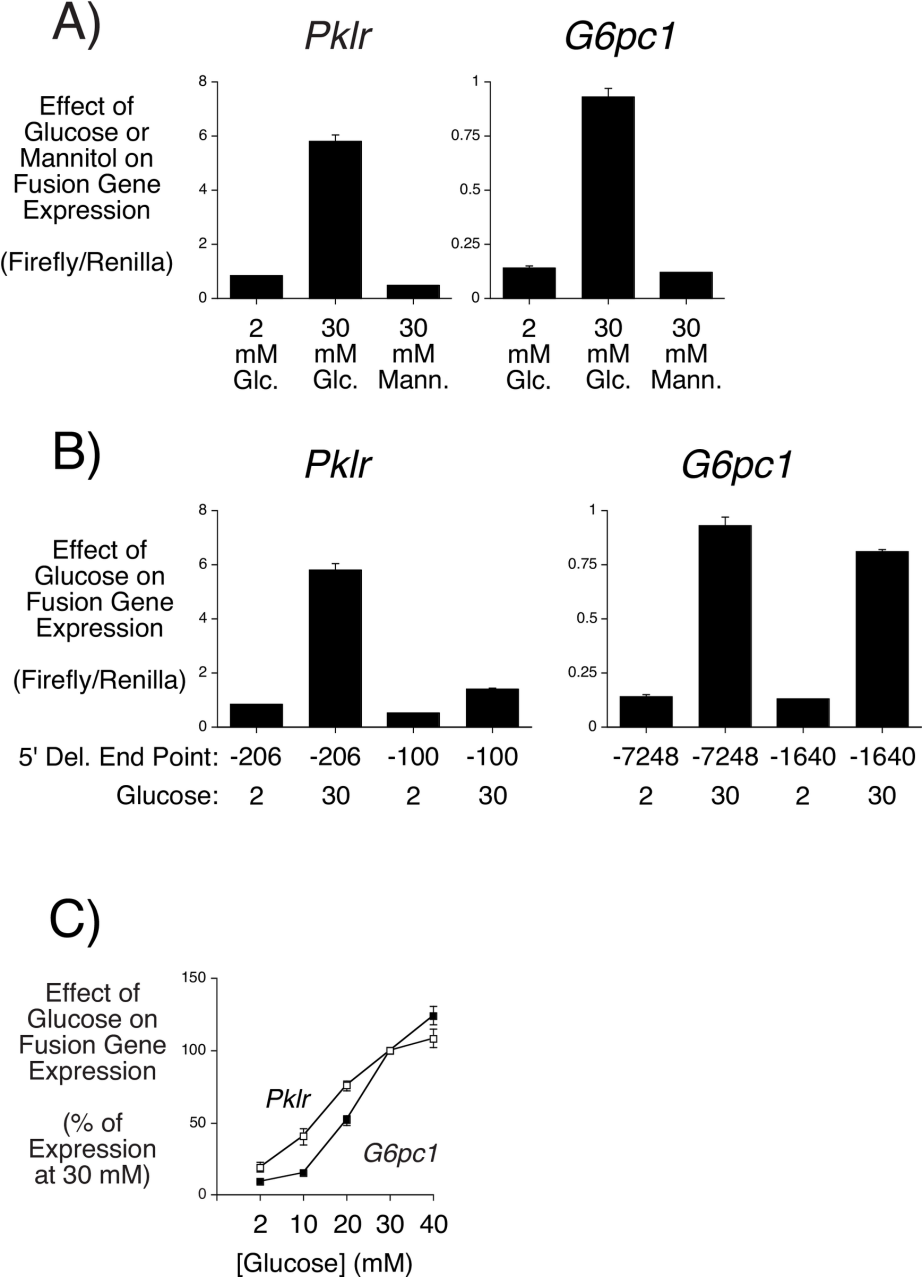
Glucose-Regulated Fusion Gene Expression in 832/13 Cells. 832/13 cells were transiently co-transfected, as described in Materials and Methods, with an expression vector encoding *Renilla* luciferase (0.5 μg) and *Pklr*-luciferase or *G6pc1*-luciferase fusion genes (2 μg) containing the promoter regions from -206 to +1 and -7253 to +66, respectively, (**Panels A** and **C**) or the indicated promoter regions (**Panel B**). Following transfection, cells were incubated for 18–20 hr in serum-free medium in the presence of the indicated concentrations of glucose (Glc) or mannitol (Mann). Cells were then harvested and luciferase activity assayed as described in Materials and Methods. Results are presented as the ratio of firefly:*Renilla* luciferase activity (**Panels A** and **B**) or a percentage of the induction achieved with 30 mM glucose (**Panel C**). Results represent the mean ± S.E.M. of 3 experiments using independent preparations of all fusion gene constructs in which each experimental condition was assayed in triplicate.

Newgard and colleagues [43,44] have previously described a highly glucose responsive INS-1 cell line variant, designated 832/13. Rat *G6pc1* [45] and liver pyruvate kinase (*Pklr*) [46] fusion gene expression are robustly induced by glucose in 832/13 cells. Fig 2A shows that, following transient transfection of 832/13 cells with luciferase fusion genes containing *Pklr* promoter sequence between -206 and +1 or *G6pc1* promoter sequence between -7248 and +62, glucose markedly stimulated reporter gene expression, confirming published reports [45,46]. Mannitol, a control for the osmotic effect of glucose, had no effect (Fig 2A). Deletion of the *Pklr* promoter region between -206 and -101 markedly reduced the effect of glucose on *Pklr*-luciferase fusion gene expression whereas deletion of the *G6pc1* promoter region between -7248 and -1641 had little effect on glucose-stimulated *G6pc1*-luciferase fusion gene expression (Fig 2B). A comparison of the EC_50_ for glucose-stimulated *Pklr*-luciferase and *G6pc1*-luciferase expression showed that *Pklr*-luciferase fusion gene expression was more sensitive to glucose (Fig 2C).

**Fig 3:**
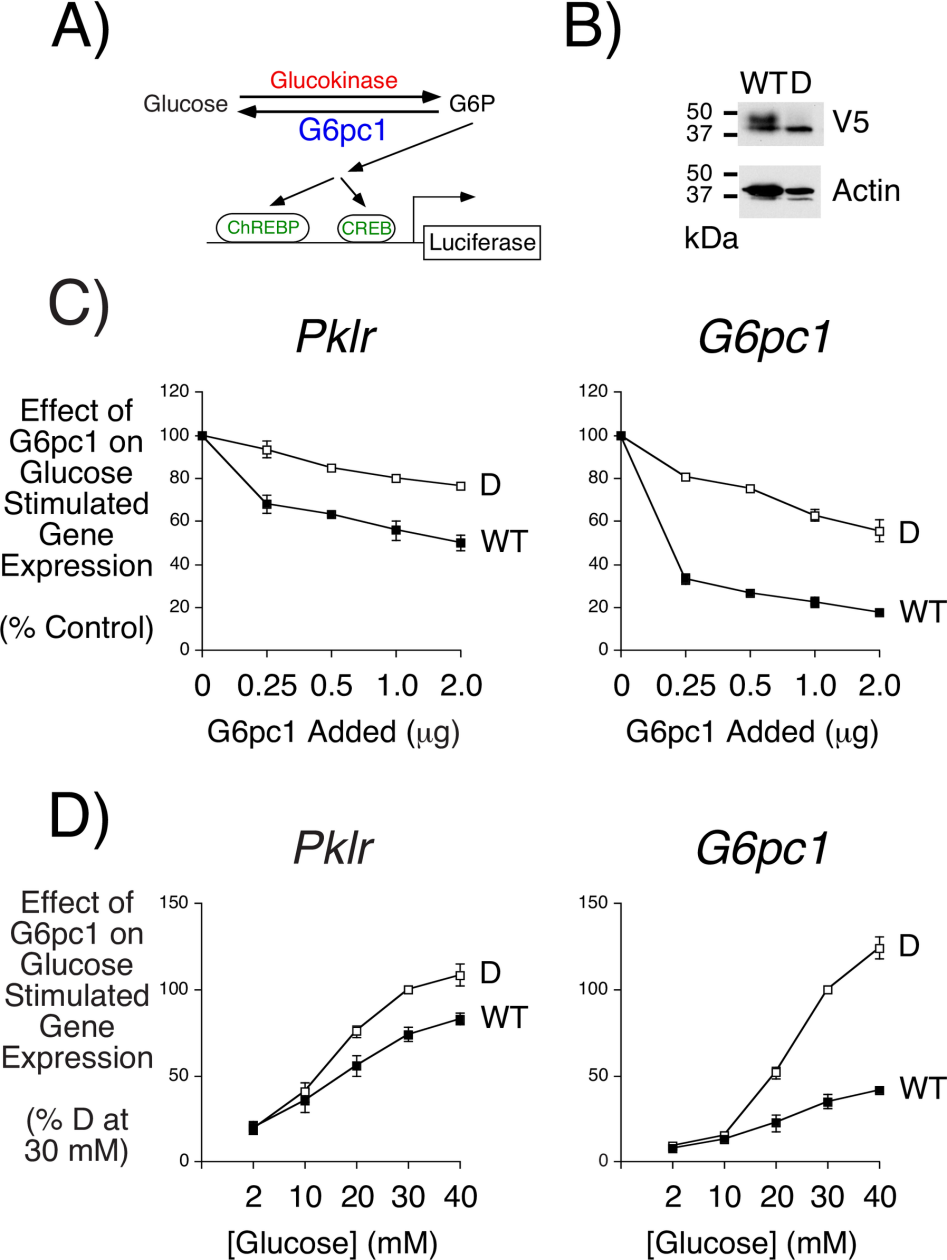
Overexpression of G6pc1 Suppresses Glucose-Stimulated Fusion Gene Expression in 832/13 Cells. **Panel A:** Schematic illustrating how the extent of glucose cycling catalyzed by glucokinase and G6pc1 determines intracellular G6P levels and hence activation of fusion gene expression through ChREBP and CREB. **Panel B:** Western blot showing that wild type (WT) and catalytically dead (D) G6pc1 are expressed at similar levels following expression in 832/13 cells. A representative blot is shown. **Panel C:** 832/13 cells were transiently co-transfected, as described in Materials and Methods, with the -206/+1 *Pklr*-luciferase or -7248/+62 *G6pc1*-luciferase fusion genes (2 μg), an expression vectors encoding *Renilla* luciferase (0.5 μg) and the indicated amounts of expression vectors encoding either wild type (WT) or catalytically dead (D) G6pc1. The total DNA added was kept constant using the empty pcDNA3 vector. Following transfection, cells were incubated for 18–20 hr in serum-free medium in the presence of 30 mM glucose. Cells were then harvested and luciferase activity assayed as described in Materials and Methods. Results were calculated as the ratio of firefly:*Renilla* luciferase activity and are presented as a percentage relative to that in 30 mM glucose-treated cells transfected with the empty pcDNA3 vector (2 μg). Results represent the mean ± S.E.M. of 3 experiments using independent preparations of both fusion gene constructs in which each experimental condition was assayed in triplicate. **Panel D:** 832/13 cells were transiently co-transfected, as described in Materials and Methods, with the -206/+1 *Pklr*-luciferase or -7248/+62 *G6pc1*-luciferase fusion genes (2 μg), an expression vectors encoding *Renilla* luciferase (0.5 μg) and 2 μg of expression vectors encoding either wild type (WT) or catalytically dead (D) G6pc1. Following transfection, cells were incubated for 18–20 hr in serum-free medium in the presence of the indicated glucose concentrations. Cells were then harvested and luciferase activity assayed as described in Materials and Methods. Results were calculated as the ratio of firefly:*Renilla* luciferase activity and are presented as a percentage relative to that in 30 mM glucose-treated cells in the presence of catalytically dead G6pc1. Results represent the mean ± S.E.M. of 3 experiments using independent preparations of both fusion gene constructs in which each experimental condition was assayed in triplicate.

Since the 832/13 cell line is derived from rat islets [43,44] these cells do not express endogenous *G6pc2* because, in contrast to all other species examined to date, *G6pc2* is a pseudogene in rats [14]. It therefore occurred to us that these cells could be used to assay glucose-6-phosphatase enzyme activity *in situ* by measuring the ability of G6pc1 expression to blunt glucose-stimulated *Pklr*-luciferase or *G6pc1*-luciferase fusion gene expression (Fig 3A). We hypothesized that G6pc1 would repress glucose-stimulated fusion gene expression by stimulating G6P hydrolysis [47], therefore opposing the action of the endogenous glucokinase in these cells [48] and thereby reducing glycolytic flux. To test this hypothesis plasmids encoding wild type (WT) G6pc1 or a catalytically dead (D) variant were co-transfected with the *Pklr*-luciferase and *G6pc1*-luciferase fusion genes. WT and catalytically dead G6pc1 were expressed at similar levels (Fig 3B). In the catalytically dead variant AA 83 was changed from arginine to alanine, which abolishes G6P hydrolysis [49]. Fig 3C and 3D show that WT G6pc1 repressed glucose-stimulated *Pklr*-luciferase and *G6pc1*-luciferase fusion gene expression relative to the expression obtained in the presence of catalytically dead G6pc1.

Most importantly, Fig 3C and 3D demonstrate that the effect of WT G6pc1 on glucose-stimulated *Pklr*-luciferase and *G6pc1*-luciferase fusion gene expression was not equivalent with G6pc1 mediating a greater repression of the latter. For the purpose of studying the impact of SNPs on glucose-6-phosphatase enzyme activity, subsequent experiments therefore examined the repression of glucose-stimulated *G6pc1*-luciferase fusion gene expression by glucose-6-phosphatase.

### Analysis of the Effect of Human *G6PC2* SNPs on the Glucose-6-Phosphatase Activity of Mouse G6pc1

**Fig 4:**
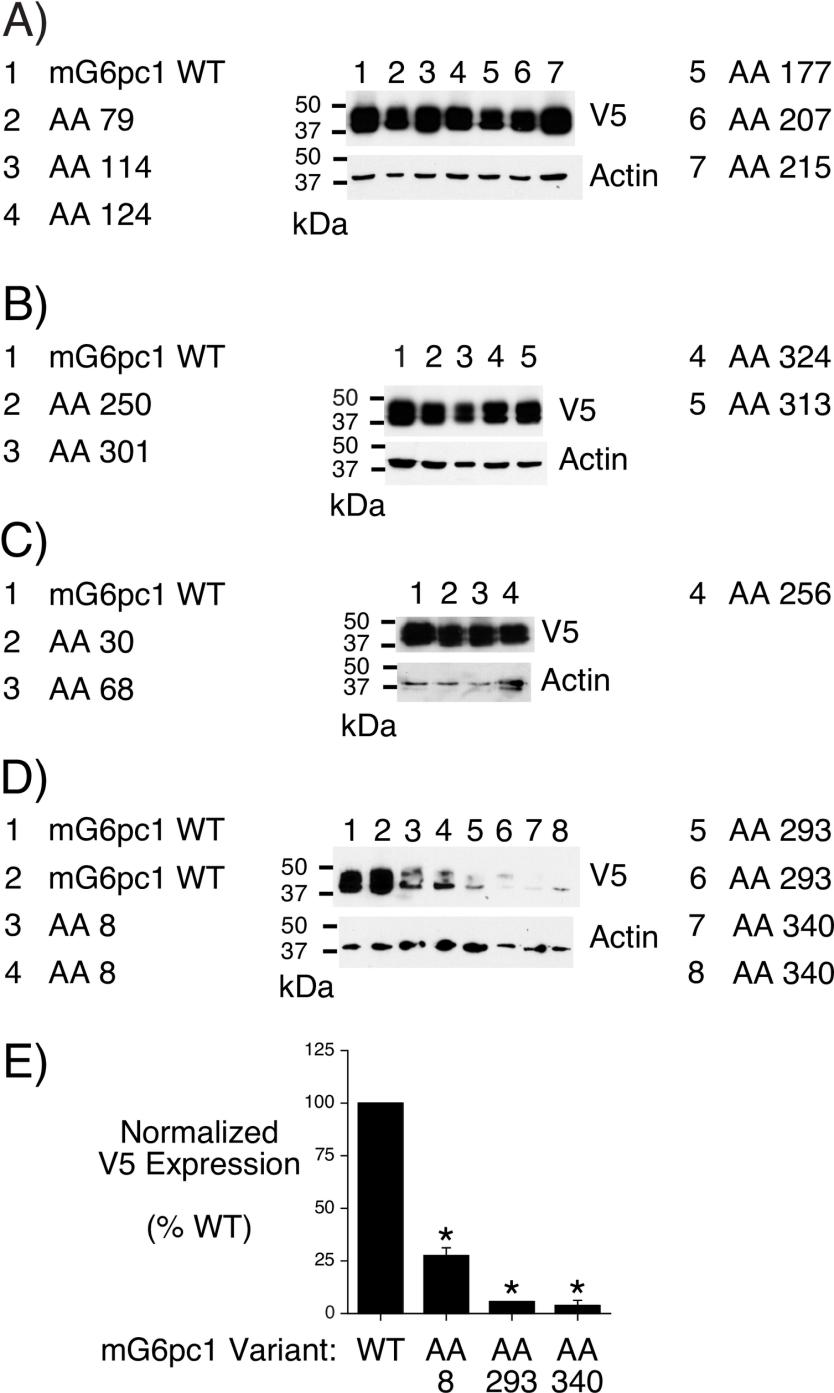
Analysis of the Effect of Amino Acid Changes on Mouse G6pc1 Protein Expression. 832/13 cells were transiently transfected, as described in Materials and Methods, with expression vectors encoding either wild type (WT) mouse (m) G6pc1 or G6pc1 variants in which the indicated amino acid (AA) had been changed as shown in Table 1. Following transfection, cells were incubated for 18–20 hr in serum-containing medium. Cells were then harvested and protein expression assayed as described in Materials and Methods. In some instances these AA changes did not affect G6pc1 protein expression (**Panels A**-**C**). In other cases they resulted in reduced expression (**Panel D**); these data were quantitated by scanning with the results in **Panel E** showing the mean ± S.E.M. of 4 experiments. For simplicity and comparison with Fig 8, these AAs are numbered based on the position of the equivalent conserved AA in human G6PC2 (Fig 1).

For these experiments we transfected 0.05 μg of plasmids encoding various G6pc1 variants, which confers a sub-maximal repression of glucose-stimulated *G6pc1*-luciferase fusion gene expression (Fig 3C). This approach allowed for the identification of both inhibitory and activating variants. Using this assay we determined that the AA changes associated with the rs144254880 (Arg79Gln), rs149663725 (Gly114Arg) and rs2232326 (Ser324Pro) SNPs markedly reduce G6pc1 enzyme activity (Table 1) without affecting protein expression (Fig 4A and 4B). For simplicity and comparison with the effect of these variants on human G6PC2 protein expression, these conserved AAs are numbered based on the position of the AA in human G6PC2 rather than their actual location in mouse G6pc1 (Fig 1). The AA changes associated with the rs142189264 (Ser30Phe), rs199682245 (Asn68Ile) and rs150538801 (Phe256Leu) SNPs also reduced G6pc1 enzyme activity (Table 1), though to a lesser degree, but again without affecting protein expression (Fig 4C). The AA changes associated with several other SNPs had statistically significant though minor effects on G6pc1 enzyme activity (Table 1), without affecting protein expression (S1 Fig). Interestingly, the AA changes associated with the rs368382511 (Gly8Glu), rs374055555 (Arg293Trp) and rs2232327 (Pro340Leu) SNPs markedly reduced G6pc1 protein expression (Fig 4D and 4E) but only the latter had a marked effect on enzyme activity (Table 1). This result suggests that these AA changes may actually be increasing the specific activity of G6pc1.

Table 1 and Fig 1 show that there are six SNPs in *G6PC2* that alter AAs that are conserved in G6PC1 and where mutation of these AAs in G6PC1 causes GSD type 1a [40]. However, these SNPs change the residue associated with GSD type 1a to an AA distinct from that that causes GSD type 1a. For example, rs372008743 changes a glutamine at residue 16 to a histidine whereas the mutation associated with GSD type 1a involves a change from a glutamine at residue 16 to an arginine (Table 1; Ref. [40]). For four of these 6 SNPs the AA change associated with the *G6PC2* SNP had little effect on G6pc1 enzyme activity or protein expression (Table 1) suggesting that the change is silent. However, for two of these 6 SNPs the AA change associated with the *G6PC2* SNP markedly affected G6pc1 enzyme activity (rs144254880; Arg79Gln) (Table 1) or expression (rs374055555; Arg293Trp) (Fig 4D and 4E).

### Analysis of the Effect of Human *G6PC2* Codon Variation on G6PC2 Protein Expression

**Fig 5:**
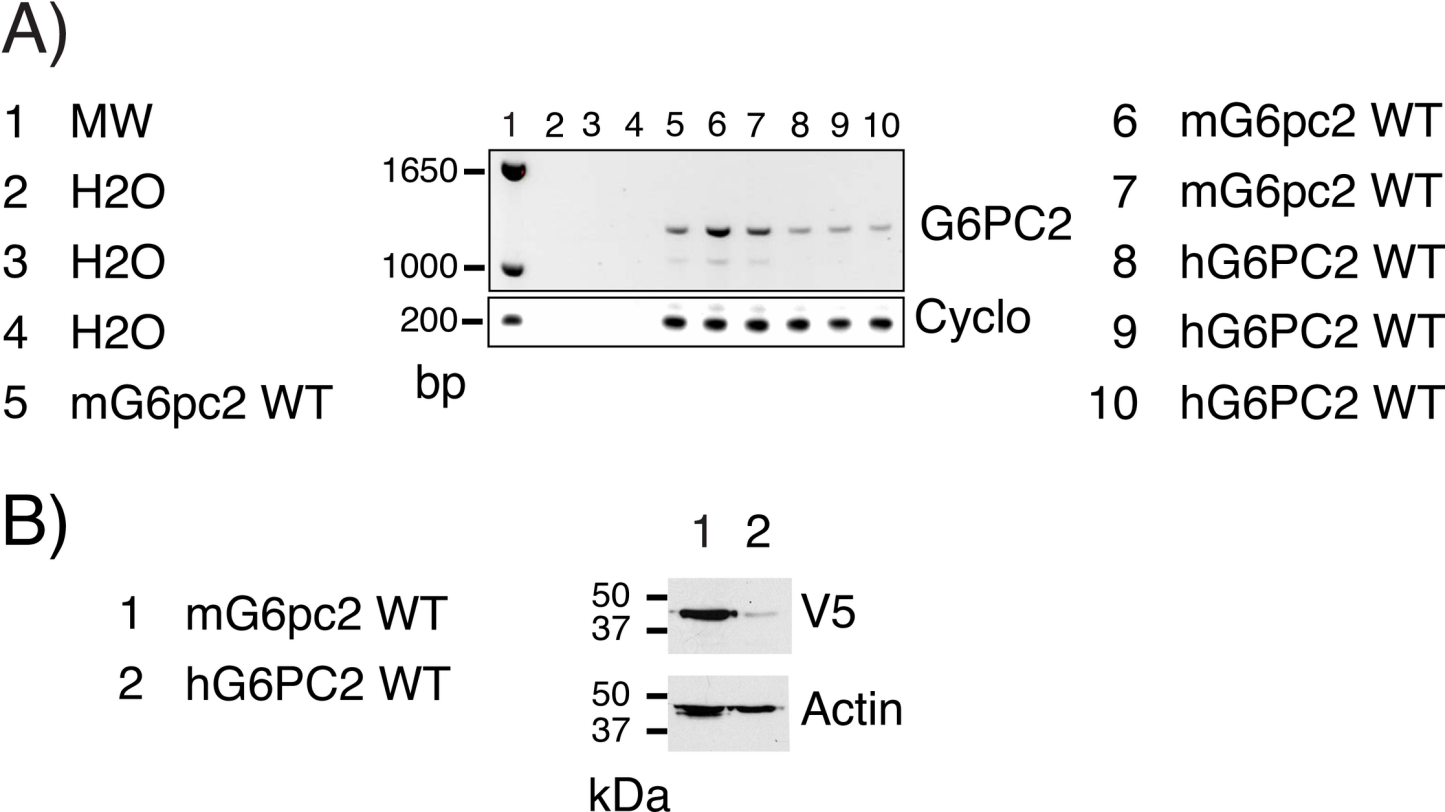
Analysis of Human *G6PC2* and Mouse *G6pc2* mRNA and Protein Expression. COS 7 cells were transiently transfected, as described in Materials and Methods, with expression vectors encoding either wild type (WT) mouse (m) G6pc2, human (h) G6PC2 or the empty pcDNA3 vector. Following transfection, cells were incubated for 18–20 hr in serum-containing medium. Cells were then harvested and either RNA (**Panel A**) or protein (**Panel B**) expression were assayed as described in Materials and Methods. A representative agarose gel (**Panel A**) or Western blot (**Panel B**) are shown. The faint band of the same size in the empty vector transfected cells represents background plasmid contamination of our PCR reagents/tubes.

Before beginning the analysis of non-synonymous human *G6PC2* SNPs on G6PC2 protein expression we sought to maximize human G6PC2 protein expression in transient transfection assays. Plasmids encoding V5 His-tagged variants of human *G6PC2* and mouse *G6pc2* [14,30,31] were transiently transfected into COS cells. Fig 5A shows that human *G6PC2* and mouse *G6pc2* RNA were expressed at similar levels but mouse G6pc2 protein expression was much higher than human G6PC2 (Fig 5B).

**Fig 6:**
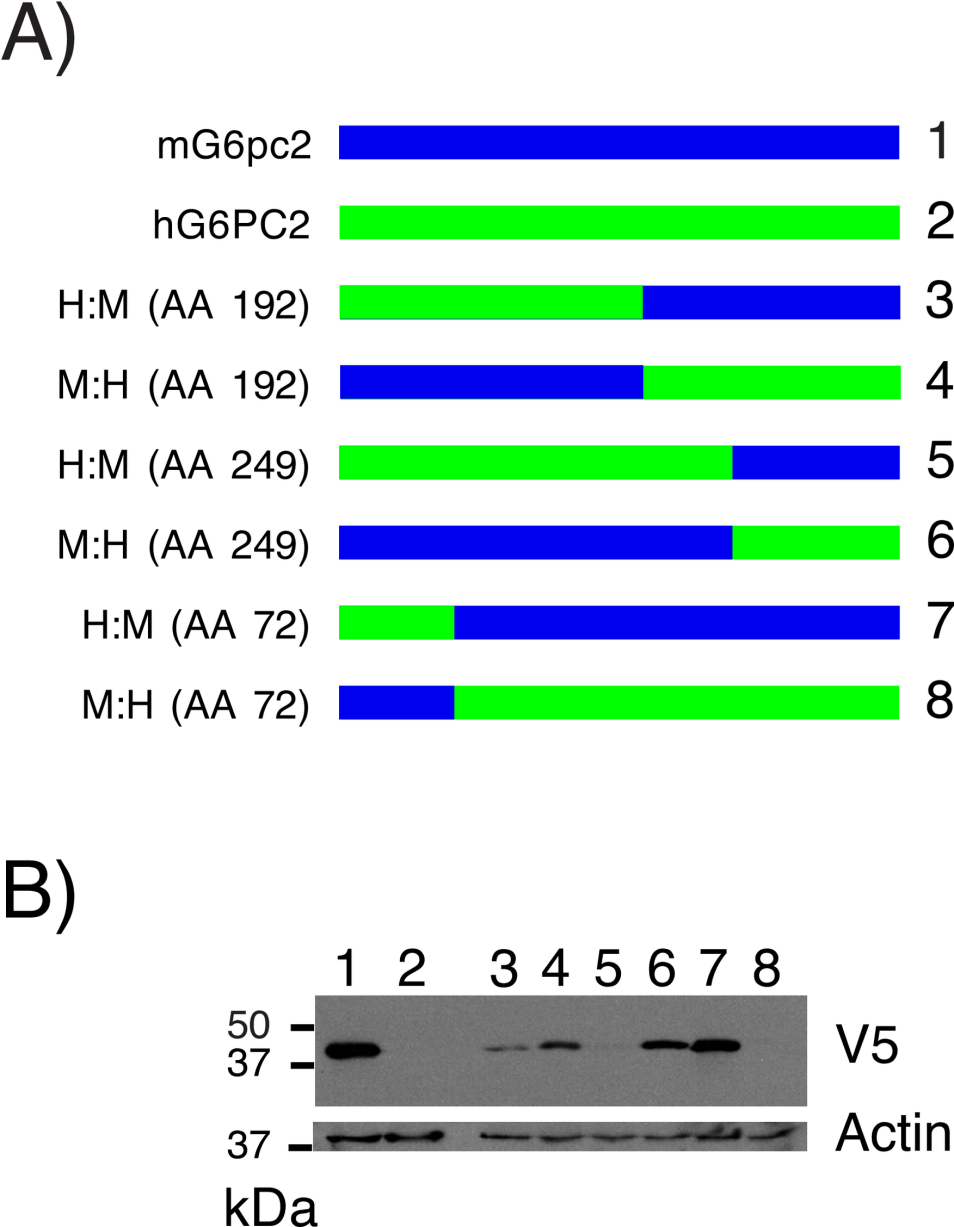
Analysis of Human G6PC2:Mouse G6pc2 Chimeric Protein Expression. 832/13 cells were transiently transfected, as described in Materials and Methods, with expression vectors encoding either wild type mouse (m) G6pc2, human (h) G6PC2 or the indicated chimeric proteins (**Panel A**). Following transfection, cells were incubated for 18–20 hr in serum-containing medium. Cells were then harvested and protein expression assayed as described in Materials and Methods (**Panel B**). A representative blot is shown.

**Fig 7:**
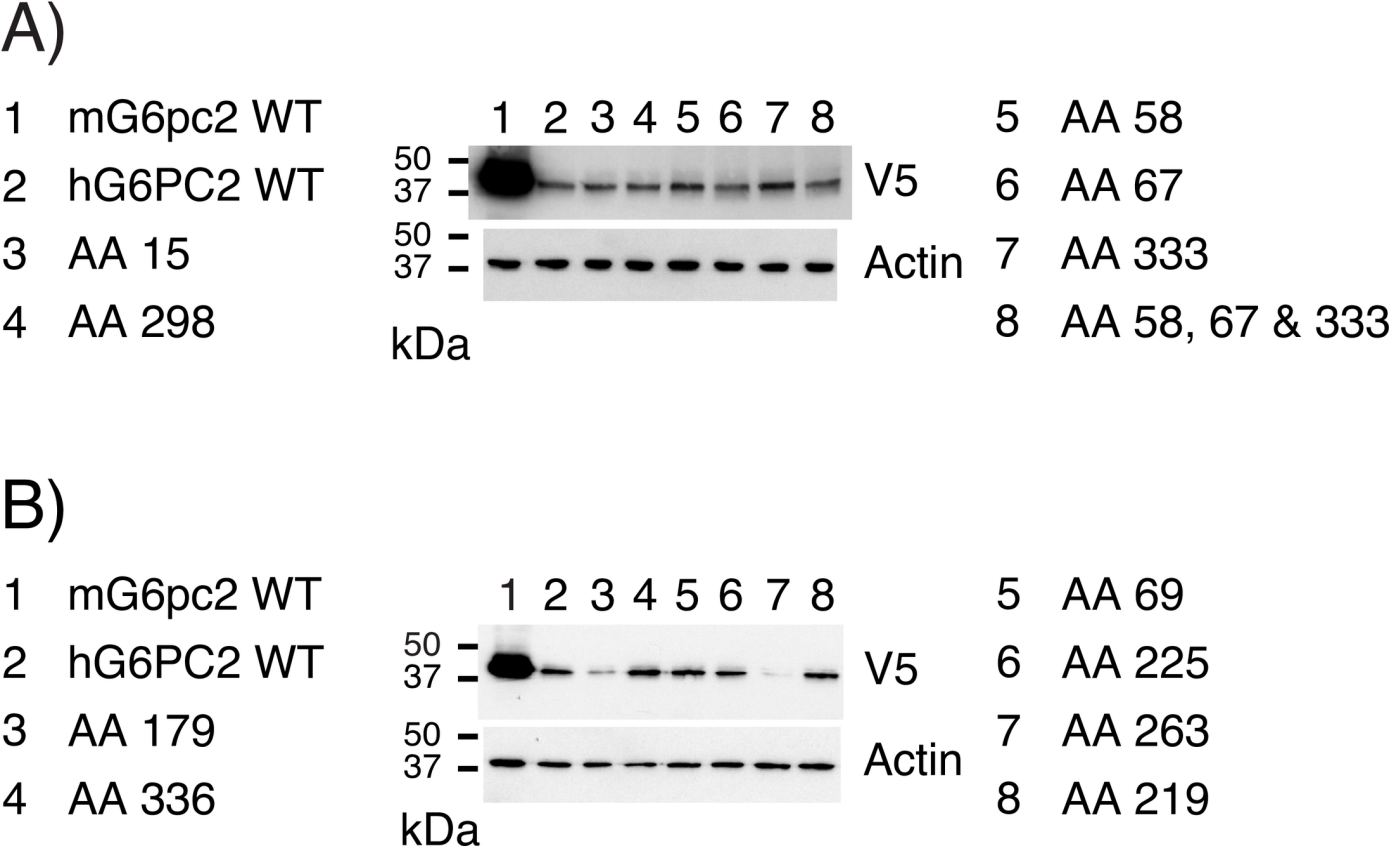
Analysis of the Effect of Human *G6PC2* Codon Variation on Protein Expression. 832/13 cells were transiently transfected, as described in Materials and Methods, with expression vectors encoding either wild type (WT) mouse (m) G6pc2, human (h) G6PC2 or G6PC2 variants in which the codon used to encode the indicated AAs had been optimized as shown in S2 Table and S3 Table. Following transfection, cells were incubated for 18–20 hr in serum-containing medium. Cells were then harvested and protein expression assayed as described in Materials and Methods. In some instances codon optimization resulted in a switch to the same codon used to encode the equivalent AA in mouse G6pc2 (**Panel A**; S2 Table). In other cases this resulted in a switch to a codon that was distinct from the codon used to encode the equivalent AA in mouse G6pc2 (**Panel B**; S3 Table). Representative blots are shown.

Chimeras of human G6PC2 and mouse G6pc2 were generated to investigate whether this difference in protein expression was associated with a particular region of the human G6PC2 or mouse G6pc2 proteins (Fig 6A). The results show that chimeric protein expression decreased as the proportion of human *G6PC2* coding sequence increased (Fig 6B). The region of G6PC2 between AAs 72 and 192 appeared to have the greatest impact on the difference in expression between mouse G6pc2 and human G6PC2 (Fig 6B). In this region 110/121 AAs are conserved between mouse G6pc2 and human G6PC2. This suggested that multiple differences between human and mouse codons potentially explained the difference in protein expression. We therefore next investigated the effect of switching individual human *G6PC2* codons that are rarely present in human mRNAs, with codons that code for the same amino acid (AA) but are more commonly found in human mRNAs. In some instances this resulted in a switch to the same codon used to encode the equivalent AA in mouse G6pc2 (Fig 7A; S2 Table). In other cases this resulted in a switch to a codon that was distinct from the codon used to encode the equivalent AA in mouse G6pc2 (Fig 7B; S3 Table). Switching the codons encoding three AAs, 58, 67 and 333, resulted in a slight improvement in human G6PC2 expression but the effect of combining these codon changes was not additive (Fig 7A). Changing two other codons, encoding AAs 179 and 263, further reduced human G6PC2 expression (Fig 7B). These data suggest that the molecular basis for the increased expression of mouse G6pc2 versus human G6PC2 is complex and involves differences in translation efficiency and/or stability that are conferred by multiple codons and/or AAs, respectively.

### Analysis of the Effect of Human *G6PC2* SNPs on G6PC2 Protein Expression

**Fig 8:**
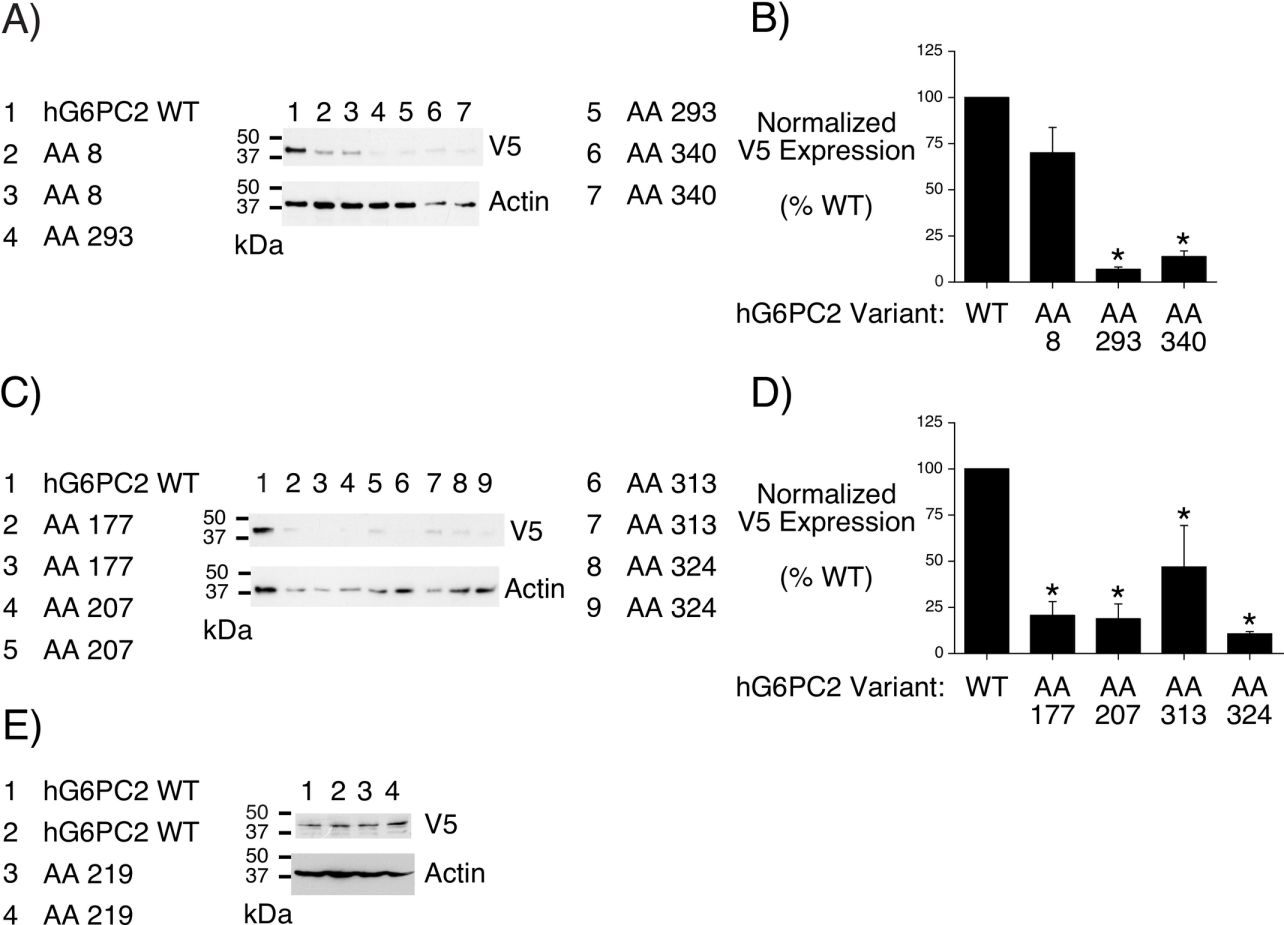
Analysis of the Effect of Human *G6PC2* SNPs on Human G6PC2 Protein Expression. COS 7 cells were transiently transfected, as described in Materials and Methods, with expression vectors encoding either wild type (WT) human (h) G6PC2 or G6PC2 variants in which the indicated amino acid (AA) had been changed as shown in Table 1. Following transfection, cells were incubated for 18–20 hr in serum-containing medium. Cells were then harvested and protein expression assayed as described in Materials and Methods. The variants shown either reduced (**Panels A-D**) or had little effect (**Panel E**) on human G6PC2 protein expression. Data were quantitated by scanning with the results in **Panels B** and **D** showing the mean ± S.E.M. of 4 experiments. Representative blots are shown.

We next analyzed the effect of several human non-synonymous *G6PC2* SNPs on human G6PC2 protein expression (Table 1). We began by analyzing the 3 SNPs that were associated with reduced mouse G6pc1 protein expression (Fig 4D and 4E), namely rs368382511 (Gly8Glu), rs374055555 (Arg293Trp) and rs2232327 (Pro340Leu). Fig 8A and 8B show that rs374055555 (Arg293Trp) and rs2232327 (Pro340Leu) also confer reduced expression of human G6PC2 in COS cells, with a trend towards reduced expression observed with rs368382511 (Gly8Glu). We next analyzed three SNPs, namely rs138726309 (His177Tyr), rs2232323 (Tyr207Ser) and rs492594 (Val219Leu) that Mahajan et al. [50] recently showed reduced human G6PC2 protein expression in 832/13 cells. Fig 8C and 8D show that rs138726309 (His177Tyr) and rs2232323 (Tyr207Ser) also confer reduced expression of human G6PC2 in COS cells. In contrast, the rs492594 (Val219Leu) variant had little effect on human G6PC2 expression in COS cells (Fig 8E). The AA changes associated with rs138726309 (His177Tyr) and rs2232323 (Tyr207Ser) did not affect mouse G6pc1 protein expression (Fig 4A). The rs492594 (Val219Leu) variant alters an AA that is not conserved in mouse G6pc2, human G6PC1 or mouse G6pc1 (S4 Table).

Finally, we analyzed two additional SNPs at the C terminus of human G6PC2, namely rs137857125 (Pro313Leu) and rs2232326 (Ser324Pro). Fig 8C and 8D show that these SNPs also confer reduced expression of human G6PC2 in COS cells. In contrast, the AA changes associated with these SNPs did not affect mouse G6pc1 protein expression (Fig 4B). Strikingly, these results suggest that despite the conservation of key AAs involved in enzyme activity between mouse G6pc1, mouse G6pc2, human G6PC1 and human G6PC2 [40,49,51] the mutation of conserved AAs has variable effects on human G6PC2 and mouse G6pc1 protein expression.

## Discussion

This study focused on 22 non-synonymous SNPs in human *G6PC2* that change AAs that are conserved between human G6PC2, mouse G6pc2, human G6PC1 and mouse G6pc1 (Table 1) (Fig 1), though database analyses identified multiple additional non-synonymous *G6PC2* SNPs that affect AAs in G6PC2 that are not conserved across all four isoforms (S4 Table). We show that the AA changes associated with the rs144254880 (Arg79Gln), rs149663725 (Gly114Arg), rs2232326 (Ser324Pro), rs142189264 (Ser30Phe), rs199682245 (Asn68Ile) and rs150538801 (Phe256Leu) SNPs reduced G6pc1 enzyme activity *in situ* (Table 1) without affecting protein expression (Fig 4A–4C). We also show that the AA changes associated with the rs368382511 (Gly8Glu), rs374055555 (Arg293Trp) and rs2232327 (Pro340Leu) SNPs markedly reduced G6pc1 protein expression (Fig 4D and 4E). Finally, we show that the rs368382511 (Gly8Glu), rs374055555 (Arg293Trp), rs2232327 (Pro340Leu), rs138726309 (His177Tyr), rs2232323 (Tyr207Ser), rs137857125 (Pro313Leu) and rs2232326 (Ser324Pro) SNPs confer reduced expression of human G6PC2 (Fig 8A–8D).

Once the challenge of achieving high human G6PC2 expression is overcome (Figs 5–7), future studies will aim to determine whether the SNPs that affect mouse G6pc1 enzyme activity also affect human G6PC2 enzyme activity. This seems highly likely since site directed mutagenesis studies [49,51] and the analysis of mutations causing GSD type 1a [40] have shown AAs that are essential for high G6PC1 enzyme activity are conserved between mouse G6pc1, mouse G6pc2, human G6PC1 and human G6PC2. Indeed, of the 56 AAs in human G6PC1 mutation of which gives rise to GSD type 1a [40], 51 are conserved or represent conserved changes in human G6PC2 (S1 Table). It is striking that the *G6PC2* SNPs rs138726309 (His177Tyr) and rs2232323 (Tyr207Ser), that have been linked to variations in FBG [50,52,53], affect AAs mutation of which in G6PC1 cause GSD type 1a (Table 1) [40]. Similarly, site directed mutagenesis studies [54] have shown a conservation of catalytically important AAs between G6PC1 and the G6PC3 isoform of glucose-6-phosphatase, initially referred to as UGRP, even though they share only a 36% overall AA conservation [30]. In humans mutations in G6PC3 cause Dursun syndrome [55,56]. Several mutations of residues that are identical between G6PC1 and G6PC3 have been associated with GSD type 1a and Dursun syndrome, respectively [55,56], supporting the notion that SNPs that alter conserved residues in all three G6PC1 isoforms will likely have similar effects on enzyme activity because catalytically important residues are conserved in all three isoforms.

Future studies will also use Vanderbilt University’s BioVU biobank to examine whether SNPs that affect human G6PC2 protein expression or activity are associated with altered phenotypic characteristics in humans, such as FBG and T2D risk. BioVU is a DNA biobank linked to a de-identified version of the Vanderbilt electronic health records, called the Synthetic Derivative (SD) [57,58]. The SD can be screened, using a procedure referred to as a PheWAS, to identify associations between specific SNPs and human diseases as well as associations with altered plasma hormone/metabolite levels [59–63]. Of particular interest will be the medical records of individuals with *G6PC2* SNPs that result in frameshift mutations or premature termination (S5 Table).

Mahajan et al. [50] recently showed that the rs138726309 (His177Tyr) and rs2232323 (Tyr207Ser) SNPs result in reduced human G6PC2 protein expression in HEK293 and INS-1E cells, which we confirmed in COS cells (Fig 8C and 8D). They also showed that another SNP rs492594 (Val219Leu), that changes an AA that is not conserved in mouse G6pc2, human G6PC1 or mouse G6pc1 (Fig 1) (S1 Table), also results in reduced human G6PC2 protein expression in HEK293 cells though not in INS-1E cells [50]. We observed that this SNP also does not appear to affect human G6PC2 protein expression in COS cells (Fig 8E). This suggests that for this particular SNP, unknown cell line-dependent factors influence its action on G6PC2 expression. As with the initially described GWAS SNP, rs560887 [8,9], Mahajan et al. [50] showed that all three of these SNPs are associated with variations in fasting plasma glucose (FPG). Horikoshi et al. [53] have also shown that the rs138726309 (His177Tyr) is associated with variations in FPG. In addition, Wessel et al. [52] have shown that rs138726309 (His177Tyr) and rs2232323 (Tyr207Ser), as well as two additional non-synonymous SNPs, rs2232326 (Ser324Pro) and rs146779637 (Arg283STOP) are associated with variations in FPG. We showed that the rs2232326 (Ser324Pro) SNP results in altered G6PC2 protein expression in COS cells (Fig 8C & 8D). Interestingly, for reasons that are unclear, Mahajan et al. [50], in contrast to Wessel et al. [52], did not observe an association between rs146779637 (Arg283STOP) and FPG. Mahajan et al. [50] speculated that the lack of association with FPG was because this variant might retain activity despite the removal of the terminal 72 AAs of G6PC2. This variant clearly merits further study, especially since the data suggest a potential difference with human G6PC1, whose activity is susceptible to C terminal truncation [49].

Previous studies have suggested a complex relationship between G6PC2 and T2D risk with apparently conflicting results in different populations [4,7,64,65]. Interestingly, Mahajan et al. [50] showed that the rs492594 (Val219Leu) SNP is associated with altered risk for T2D. In contrast, in their study of non-synonymous *G6PC2* SNPs, Wessel et al. [52] reported no association between *G6PC2* and T2D risk. The rs492594 (Val219Leu) G6PC2 variant was not included in the studies of Wessel et al. [52] so potential reasons for this apparent discrepancy between G6PC2 variation and T2D risk remain unclear. These results may indicate that the rs492594 (Val219Leu) G6PC2 variant has a unique effect on beta cell function, unrelated to the control of glycolytic flux, especially since our results (Fig 8) and the results of Mahajan et al. [50] suggest that the rs492594 (Val219Leu) variant reduces G6PC2 protein expression less than the rs138726309 (His177Tyr) and rs2232323 (Tyr207Ser) variants that were included in the studies of Wessel et al. [52]. Indeed, we have previously speculated that G6PC2 may affect beta cell endoplasmic reticulum calcium retention, in addition to its action on glycolytic flux [11]. Indirect support for such a function for G6PC2 was recently suggested by the observation that deletion of the *sorcin* gene, which regulates endoplasmic reticulum calcium retention, resulted in elevated *G6pc2* expression [66].

Our study also describes a novel assay for the measurement of glucose-6-phosphatase activity *in situ* (Figs 2 and 3). G6pc1 is unstable [67] and much remains unknown about the factors regulating G6pc1 activity [42] so this assay has the advantage that G6pc1 activity can be studied in an endogenous environment rather than in isolated and/or permeabilized microsomes. However, there are several caveats associated with this assay. Firstly, even though the amount of G6pc1 expressed was sufficient to achieve a sub-maximal repression of glucose-stimulated *G6pc1*-luciferase gene expression (Fig 3), this assay will not have the same linearity relative to an *in vitro* assay given the influence of other intracellular factors on G6pc1 activity. Secondly, in this assay apparent changes in G6pc1 activity could arise indirectly due to a change in sub-cellular distribution. Finally, because G6pc1 is located in the endoplasmic reticulum with its active site directed towards the lumen, the glucose-6-phosphatase activity of G6pc1 *in situ* is dependent on transport of its substrate G6P into the lumen by a G6P/Pi transporter, encoded by the *SLC37A4* gene [11,17]. Pan et al. [68] have shown that G6PC1 and SLC37A4 are functionally coupled. Therefore, AA changes that affect this coupling will also appear to affect the inherent glucose-6-phosphatase activity of G6pc1 in the *in situ* assay. Future studies comparing the activity of specific G6pc1 variants in this *in situ* assay and the standard *in vitro* assay may lead to the identification of variants that affect aspects of G6pc1 function other than G6P hydrolysis.

In the course of developing our novel assay we made several interesting observations about glucose-regulated gene expression. Collier et al. [46] demonstrated that the effect of glucose on rat *Pklr* gene expression in 832/13 cells is mediated by the carbohydrate response element binding protein (ChREBP), which binds a carbohydrate response element (ChoRE) located between -188 and -172 in the rat *Pklr* promoter [69]. Consistent with this observation, deletion of the promoter region between -206 and -101 markedly reduced the effect of glucose on *Pklr*-luciferase fusion gene expression (Fig 2B). In contrast, Pederson et al. [45] demonstrated that the effect of glucose on rat *G6pc1* expression in 832/13 cells is mediated by two promoter elements, a ChoRE located between -3616 and -3600 that binds ChREBP [45], and a cAMP response element (CRE) located between -163 and -156 that binds CRE binding protein (CREB) [70]. Pederson et al. [45] found that deletion of the G6PC1 ChoRE reduced the glucose response by ~80%. However, Fig 2B shows that deletion of the promoter region between -7248 and -1641 had little effect on glucose-stimulated *G6pc1*-luciferase fusion gene expression, suggesting that differences possibly related to cell passage number or growth conditions have altered the relative importance of ChREBP and CREB in glucose signaling to the *G6pc1* promoter in our 832/13 cells. Interestingly, *Pklr*-luciferase fusion gene expression was more sensitive to glucose suggesting that the signaling pathways used by glucose to regulate ChREBP and CREB are distinct (Fig 2C). Consistent with this idea, the effect of WT G6pc1 on glucose-stimulated *Pklr*-luciferase and *G6pc1*-luciferase fusion gene expression was not equivalent with G6pc1 mediating a greater repression of the latter (Fig 3C and 3D). This result not only suggests that the glucose-signaling pathways to ChREBP and CREB are distinct but that G6pc1 preferentially influences the latter. Finally, we also observed that catalytically dead G6pc1 partially represses glucose-stimulated fusion gene expression (Fig 3C and 3D). This may reflect activation of the endoplasmic reticulum stress response [71] and could also explain why catalytically dead G6pc1 has a greater effect on *G6pc1* versus *Pklr* fusion gene expression since the former promoter contains a stress response element [72]. Interestingly, over expression of G6PC2 in mice causes diabetes due to activation of the ER stress response [73].

## Supporting Information

S1 Fig Analysis of the Effect of Amino Acid Changes on Mouse G6pc1 Protein Expression. 832/13 cells were transiently transfected, as described in Materials and Methods, with expression vectors encoding either wild type (WT) mouse (m) G6pc1 or G6pc1 variants in which the indicated amino acid (AA) had been changed as shown in Table 1. Following transfection, cells were incubated for 18–20 hr in serum-containing medium. Cells were then harvested and protein expression assayed as described in Materials and Methods. With the exception of AA 8, these AA changes did not markedly affect G6pc1 protein expression. Representative blots are shown. For simplicity and comparison with Fig 8, these AAs are numbered based on the position of the equivalent AA in human G6PC2 (Fig 1).
(PDF)

S1 Table Amino Acids in Human G6PC1 Whose Mutation Causes Glycogen Storage Disease Type 1a are Highly Conserved in Mouse G6pc1, Mouse G6pc2 and Human G6PC2. The Table shows AAs in human G6PC1 whose mutation causes glycogen storage disease (GSD) type 1a [40] and whether these AAs are conserved or similar in mouse G6pc1, mouse G6pc2 and human G6PC2.
(PDF)

S2 Table Comparison of Codon Usage in Human *G6PC2* mRNA with Common Codon Usage in Human mRNAs. The Table shows that the codons used to encode the indicated AAs in human G6PC2 are not the most commonly used codons to encode these AAs in human proteins. The Table also shows that the codons that are commonly used to encode these AAs in human proteins are the same as the codons used to encode these AAs in mouse G6pc2. The effect on human G6PC2 protein expression of changing these codons to the most frequently used codon was assessed as described in Fig 7A. Codon usage in human mRNAs is described at the following website: http://www.kazusa.or.jp/codon/cgi-bin/showcodon.cgi?species=9606&aa=1&style=N
(PDF)

S3 Table Comparison of Codon Usage in Human *G6PC2* mRNA with Common Codon Usage in Human mRNAs. The Table shows that the codons used to encode the indicated AAs in human G6PC2 are not the most commonly used codons to encode these AAs in human proteins. The Table also shows that the codons that are commonly used to encode these AAs in human proteins are also different to the codons used to encode these AAs in mouse G6pc2. The effect on human G6PC2 protein expression of changing these codons to the most frequently used codon was assessed as described in Fig 7B. In this analysis we just focused on codons for AAs that are conserved between mouse G6pc2 and human G6PC2. In other words, we did not optimize codons that encode AAs that are unique to human G6PC2.
(PDF)

S4 Table Human *G6PC2* SNPs that Alter Amino Acids that are not Conserved in Human G6PC2, Mouse G6pc2, Human G6pc and Mouse G6pc. Human *G6PC2* SNPs that change AAs that are not uniformly conserved in human G6PC2, mouse G6pc2, human G6PC1 and mouse G6pc1 were identified using the UCSC Genome Browser (https://genome.ucsc.edu/) and HumSAVR (http://omictools.com/humsavar-tool) databases. The G6PC2 domain affected by each AA change was predicted by comparison with the proposed structure of G6PC1 [41]. **, this residue has been associated with variations in FBG in healthy individuals who do not have diabetes [50].
(PDF)

S5 Table Human *G6PC2* SNPs that Cause Frameshift Mutations and Premature Termination. Human *G6PC2* SNPs that result in frameshift mutations or premature termination were identified using the UCSC Genome Browser (https://genome.ucsc.edu/) and HumSAVR (http://omictools.com/humsavar-tool) databases. The rs35259259 (Gly186Ala) SNP represents a deletion of a single base pair (G) at the *G6PC2* exon five 5’ splice junction causing a shift in the open reading frame. The G6PC2 domain affected by each AA change was predicted by comparison with the proposed structure of G6PC1 [41].
(PDF)
