# Supplementary figures and images for: Functional Analysis of Mouse G6pc1 Mutations Using a Novel In Situ Assay for Glucose-6-Phosphatase Activity and the Effect of Mutations in Conserved Human G6PC1/G6PC2 Amino Acids on G6PC2 Protein Expression
Source: PLoS One. 2016 Sep 9;11(9):e0162439. doi: 10.1371/journal.pone.0162439 (PMC5017610; doi:10.1371/journal.pone.0162439)

# S1 Fig

A)

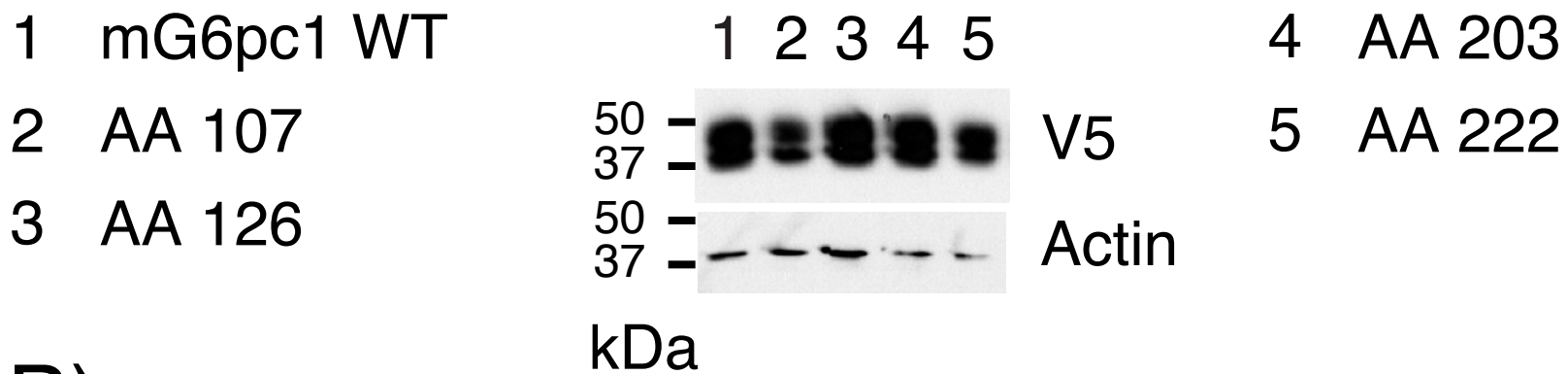

B)

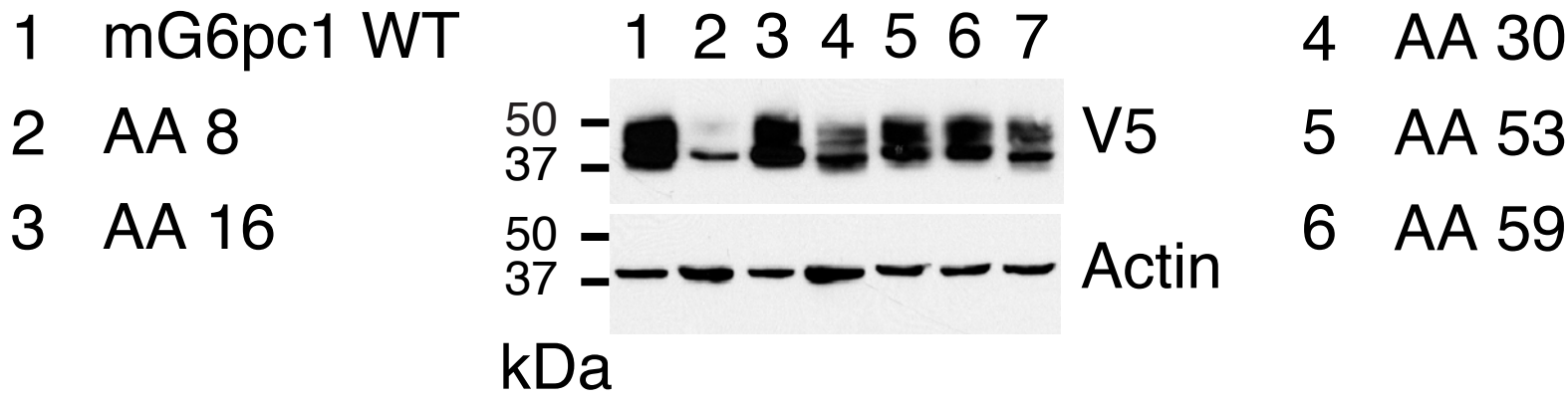

Supplement: S1 Fig — 832/13 cells were transiently transfected, as described in Materials and Methods, with expression vectors encoding either wild type (WT) mouse (m) G6pc1 or G6pc1 variants in which the indicated amino acid (AA) had been changed as shown in Table 1. Following transfection, cells were incubated for 18–20 hr in serum-containing medium. Cells were then harvested and protein expression assayed as described in Materials and Methods. With the exception of AA 8, these AA changes did not markedly affect G6pc1 protein expression. Representative blots are shown. For simplicity and comparison with Fig 8, these AAs are numbered based on the position of the equivalent AA in human G6PC2 (Fig 1). (PDF) [file pone.0162439.s001.pdf]
