# Supplementary material for: Functional Analysis of Mouse G6pc1 Mutations Using a Novel In Situ Assay for Glucose-6-Phosphatase Activity and the Effect of Mutations in Conserved Human G6PC1/G6PC2 Amino Acids on G6PC2 Protein Expression
Source: PLoS One. 2016 Sep 9;11(9):e0162439. doi: 10.1371/journal.pone.0162439 (PMC5017610; doi:10.1371/journal.pone.0162439)
Supplement: S1 Table — The Table shows AAs in human G6PC1 whose mutation causes glycogen storage disease (GSD) type 1a [40] and whether these AAs are conserved or similar in mouse G6pc1, mouse G6pc2 and human G6PC2. (PDF) [file pone.0162439.s002.pdf]

S1 Table

hG6PC1 GSD1a Mutations	mG6pc1		mG6pc2		hG6PC2	
AA change	AA	Conserved	AA	Conserved	AA	Conserved
M5R	M	yes	M	yes	M	yes
T16A	T	yes	I	no	I	no
T16R	T	yes	I	no	I	no
Q20R	Q	yes	Q	yes	Q	yes
D38V	D	yes	D	yes	D	yes
Q54P	K	similar	N	no	N	no
W63R	W	yes	W	yes	W	yes
A65P	A	yes	A	yes	A	yes
G68R	G	yes	G	yes	G	yes
K76N	K	yes	K	yes	K	yes
W77R	W	yes	W	yes	W	yes
G81R	G	yes	G	yes	G	yes
R83C	R	yes	R	yes	R	yes
R83H	R	yes	R	yes	R	yes
T108I	T	yes	T	yes	T	yes
E110K	E	yes	E	yes	E	yes
E110Q	E	yes	E	yes	E	yes
T111I	T	yes	T	yes	T	yes
P113L	P	yes	P	yes	P	yes
H119L	H	yes	H	yes	H	yes
G122D	G	yes	G	yes	G	yes
A124T	A	yes	S	similar	S	similar
W156L	W	yes	W	yes	W	yes
V166A	V	yes	V	yes	V	yes
V166G	V	yes	V	yes	V	yes
R170Q	R	yes	R	yes	R	yes
L173P	L	yes	I	similar	I	similar
F177C	F	yes	F	yes	F	yes
P178A	P	yes	P	yes	P	yes
P178S	P	yes	P	yes	P	yes
H179P	H	yes	H	yes	H	yes
G184E	G	yes	G	yes	G	yes
G184V	G	yes	G	yes	G	yes
G188D	G	yes	G	yes	G	yes
G188R	G	yes	G	yes	G	yes
G188S	G	yes	G	yes	G	yes
Y209C	Y	yes	Y	yes	Y	yes
L211P	L	yes	K	no	K	no
G222R	G	yes	G	yes	G	yes
G222R	G	yes	G	yes	G	yes
W236R	W	yes	W	yes	W	yes
A241T	A	yes	A	yes	A	yes
T255I	T	yes	S	similar	T	yes
P257L	P	yes	P	yes	P	yes
N264K	N	yes	N	yes	N	yes
L265P	L	yes	L	yes	L	yes
G266V	G	yes	G	yes	G	yes
G270R	G	yes	G	yes	G	yes

G270V	G	yes	G	yes	G	yes
R295C	R	yes	R	yes	R	yes
S298P	C	no	C	no	C	no
F322L	F	yes	F	yes	F	yes
V338F	V	yes	V	yes	V	yes
I341N	I	yes	I	yes	I	yes
L345R	L	yes	V	similar	V	similar
L345R	L	yes	V	similar	V	similar
