# Supplementary material for: Functional Analysis of Mouse G6pc1 Mutations Using a Novel In Situ Assay for Glucose-6-Phosphatase Activity and the Effect of Mutations in Conserved Human G6PC1/G6PC2 Amino Acids on G6PC2 Protein Expression
Source: PLoS One. 2016 Sep 9;11(9):e0162439. doi: 10.1371/journal.pone.0162439 (PMC5017610; doi:10.1371/journal.pone.0162439)
Supplement: S2 Table — The Table shows that the codons used to encode the indicated AAs in human G6PC2 are not the most commonly used codons to encode these AAs in human proteins. The Table also shows that the codons that are commonly used to encode these AAs in human proteins are the same as the codons used to encode these AAs in mouse G6pc2. The effect on human G6PC2 protein expression of changing these codons to the most frequently used codon was assessed as described in Fig 7A. Codon usage in human mRNAs is described at the following website: http://www.kazusa.or.jp/codon/cgi-bin/showcodon.cgi?species=9606&aa=1&style=N (PDF) [file pone.0162439.s003.pdf]

S2 Table

AA#	Human *G6PC2* Codon	Frequency per 1000 Human cDNAs	Most Frequently Used Codon	Mouse *G6pc2* Codon	Frequency per 1000 Human cDNAs	Frequency Difference	Effect on hG6PC2 Expression
333	CTA	7.15	CTG	CTG	39.64	32.49	Increased
15	TTG	12.93	CTG	CTG	39.64	26.71	N.C.
298	TTG	12.93	CTG	CTG	39.64	26.71	Decreased
301	TTG	12.93	CTG	CTG	39.64	26.71	Decreased
153	CTT	13.19	CTG	CTG	39.64	26.45	N.C.
48	CAA	12.34	CAG	CAG	34.23	21.89	Decreased
178	CAA	12.34	CAG	CAG	34.23	21.89	Decreased
183	GTA	7.08	GTG	GTG	28.12	21.04	N.C.
336	GTT	11.03	GTG	GTG	28.12	17.09	N.C.
289	ACA	15.11	AAG	AAG	31.86	16.75	N.C.
58	ATA	7.49	ATC	ATC	20.82	13.33	Increased
238	ATA	7.49	ATC	ATC	20.82	13.33	N.C.
67	TTA	7.67	TTC	TTC	20.28	12.61	Increased
108	GGT	10.75	GGC	GGC	22.22	11.47	N.C.
77	GGT	10.75	GGC	GGC	22.22	11.47	N.C.
