# Supplementary material for: Functional Analysis of Mouse G6pc1 Mutations Using a Novel In Situ Assay for Glucose-6-Phosphatase Activity and the Effect of Mutations in Conserved Human G6PC1/G6PC2 Amino Acids on G6PC2 Protein Expression
Source: PLoS One. 2016 Sep 9;11(9):e0162439. doi: 10.1371/journal.pone.0162439 (PMC5017610; doi:10.1371/journal.pone.0162439)
Supplement: S3 Table — The Table shows that the codons used to encode the indicated AAs in human G6PC2 are not the most commonly used codons to encode these AAs in human proteins. The Table also shows that the codons that are commonly used to encode these AAs in human proteins are also different to the codons used to encode these AAs in mouse G6pc2. The effect on human G6PC2 protein expression of changing these codons to the most frequently used codon was assessed as described in Fig 7B. In this analysis we just focused on codons for AAs that are conserved between mouse G6pc2 and human G6PC2. In other words, we did not optimize codons that encode AAs that are unique to human G6PC2. (PDF) [file pone.0162439.s004.pdf]

S3 Table

AA#	Human *G6PC2* Codon	Frequency per 1000 Human cDNAs	Most Frequently Used Codon	Mouse *G6pc2* Codon	Frequency per 1000 Human cDNAs	Frequency Difference	Effect on hG6PC2 Expression
219	CTT	13.19	CTG	CTC	39.64	26.45	N.C.
263	CTT	13.19	CTG	CTC	39.64	26.45	Decreased
225	CTT	13.19	CTG	CTC	39.64	26.45	N.C.
69	CTT	13.19	CTG	CTC	39.64	26.45	N.C.
179	GTT	11.03	GTG	GTC	28.12	17.09	Decreased
11	ATA	7.49	ATC	ATT	20.82	13.33	N.C.
303	ATA	7.49	ATC	ACA	20.82	13.33	N.C.
