# Supplementary material for: Functional Analysis of Mouse G6pc1 Mutations Using a Novel In Situ Assay for Glucose-6-Phosphatase Activity and the Effect of Mutations in Conserved Human G6PC1/G6PC2 Amino Acids on G6PC2 Protein Expression
Source: PLoS One. 2016 Sep 9;11(9):e0162439. doi: 10.1371/journal.pone.0162439 (PMC5017610; doi:10.1371/journal.pone.0162439)
Supplement: S4 Table — Human G6PC2 SNPs that change AAs that are not uniformly conserved in human G6PC2, mouse G6pc2, human G6PC1 and mouse G6pc1 were identified using the UCSC Genome Browser (https://genome.ucsc.edu/) and HumSAVR (http://omictools.com/humsavar-tool) databases. The G6PC2 domain affected by each AA change was predicted by comparison with the proposed structure of G6PC1 [41]. **, this residue has been associated with variations in FBG in healthy individuals who do not have diabetes [50]. (PDF) [file pone.0162439.s005.pdf]

S4 Table

hG6PC2 SNP	Base #	AA#	AA Conserved in mG6pc2	AA Conserved in mG6pc1	AA Conserved in hG6PC1	Domain Location
rs143670077	CGA151CAA	Arg20Gln	x			N terminus
rs34725343	TTT174GTT	Phe28Val	x			N terminus
rs375350062	AAT184AGT	Asn31Ser	x			In membrane 1
rs141321853	AGT375GGT	Ser95Gly	x			In loop
rs200209268	AAC724AGC	Asn211Ser	x			In membrane 6
rs146233425	TCC1081TAC	Ser330Tyr	x			In membrane 9
rs371294159	ACA247ATA	Thr52Ile		x		In loop
rs184807114	GCA448GTA	Ala119Val		x		In loop
rs191279338	GCA447ACA	Ala119Thr		x		In loop
rs182708685	CTC682ATC	Leu228Ile		x	x	In loop
rs182708685	CTC682GTC	Leu228Val		x	x	In loop
rs182708685	CTC774TTC	Leu228Phe		x	x	In membrane 6
rs200336133	CTC1020TTC	Leu310Phe		x	x	In membrane 8
rs201561079	ATT280ACT	Ile63Thr	x		x	In membrane 2
rs377582119	CAA691CGA	Gln200Arg				In loop
rs492594	GTT657CTT	Val219Leu**				In loop
rs371639677	AAC778ATC	Asn229Ile				In loop
rs143686780	ACA958AAA	Thr289Lys				In loop
rs373196602	CAG1024CGG	Gln311Arg				In membrane 8
rs2232328	TCT1117TGT	Ser342Cys				In membrane 9
rs367851926	AGC1141AAC	Ser350Asn				C terminus
