# Supplementary material for: Functional Analysis of Mouse G6pc1 Mutations Using a Novel In Situ Assay for Glucose-6-Phosphatase Activity and the Effect of Mutations in Conserved Human G6PC1/G6PC2 Amino Acids on G6PC2 Protein Expression
Source: PLoS One. 2016 Sep 9;11(9):e0162439. doi: 10.1371/journal.pone.0162439 (PMC5017610; doi:10.1371/journal.pone.0162439)
Supplement: S5 Table — Human G6PC2 SNPs that result in frameshift mutations or premature termination were identified using the UCSC Genome Browser (https://genome.ucsc.edu/) and HumSAVR (http://omictools.com/humsavar-tool) databases. The rs35259259 (Gly186Ala) SNP represents a deletion of a single base pair (G) at the G6PC2 exon five 5’ splice junction causing a shift in the open reading frame. The G6PC2 domain affected by each AA change was predicted by comparison with the proposed structure of G6PC1 [41]. (PDF) [file pone.0162439.s006.pdf]

S5 Table

hG6PC2 SNP	Base #	AA#	AA Conserved in mG6pc2	AA Conserved in mG6pc1	AA Conserved in hG6PC1	Domain Location
rs35259259	GGC649G	Gly186Ala	x	x	x	In membrane 5
rs143855780	CGA327TGA	Arg79STOP	x	x	x	In membrane 2
rs199741621	TGG338TGA	Trp82STOP	x	x	x	In membrane 2
rs201323443	TAT1055TAG	Tyr321STOP	x	x	x	In membrane 9
rs146779637	CGA939TGA	Arg283STOP				In loop
